# Cross-comparison study of three ELISA methodologies to measure *Shigella Sonnei* O-antigen serum IgG

**DOI:** 10.1128/msphere.00356-25

**Published:** 2025-09-15

**Authors:** Paul Stickings, Caroline Vipond, Peter Rigsby, Francesca Micoli, Omar Rossi, Francesca Mancini, Valentino Conti, Dani Cohen, Anya Bialik, Shiri Meron-Sudai, Valeria Asato, Kristen A. Clarkson, Calman A. MacLennan, Robert W. Kaminski

**Affiliations:** 1 Medicines and Healthcare Products Regulatory Agency 9059 , South Mimms, United Kingdom; 2 GSK Vaccines Institute for Global Health 622628 , Siena, Italy; 3 Department of Epidemiology and Preventive Medicine, School of Public Health, Faculty of Medical and Health Sciences, Tel Aviv University 26745, Tel Aviv, Israel; 4 Department of Diarrheal Disease Research, Bacterial Diseases Branch, Walter Reed Army Institute of Research 8394, , Silver Spring, Maryland, USA; 5 Enteric & Diarrheal Diseases, Gates Foundation 11037, Seattle, Washington, USA; Vanderbilt University Medical Center, Nashville, Tennessee, USA

**Keywords:** *Shigella*, *sonnei*, immunoassays

## Abstract

Measurement of serum immunoglobulin G (IgG) to *Shigella* lipopolysaccharide (LPS) has been proposed as a correlate of protection against shigellosis, the confirmation of which in the target population of infants in low- and middle-income countries will facilitate vaccine development and licensure. Data obtained from *Shigella sonnei* vaccine clinical studies have identified a protective serum LPS IgG threshold value that is assay-specific. In the interim period before an International Standard serum was available, a retrospective bridging study using a panel of human serum samples had been conducted to estimate the anti-*S*. *sonnei* LPS IgG levels in two ELISA methods, employed in several ongoing or recently completed age-descending *Shigella* vaccine clinical studies that correspond to the threshold titer of 1,600 established using the Tel Aviv University ELISA method. Each lab performed its own in-house ELISA protocol and tested a panel of 32 human serum samples, covering a range of anti-*S*. *sonnei* LPS IgG levels. We observed excellent correlation of the ELISA data between the laboratories and used the fitted equations from regression analysis to determine the values that correspond to an endpoint titer of 1,600 in the Tel Aviv University ELISA method. Our analysis provides a way to meaningfully compare results obtained with clinical samples across the ELISA methods used by the three participating laboratories.

**IMPORTANCE:**

To support large-scale efficacy trials, especially where efficacy trials are not feasible, the ability to compare immune response data across candidate *Shigella* vaccines can be very valuable for identifying the most promising vaccine platform and immunobridging to other populations, vaccine formulations, or additional platforms in the future. However, international standards for antibody assays are not yet available for *Shigella* vaccines currently in clinical development. Lack of standardization of *Shigella* immunoassays means that the results of antibody measurement in clinical samples from different vaccine trials or those from seroepidemiology studies cannot be easily compared. The results from this study will facilitate the comparison of immunological titers obtained across different *Shigella* vaccine studies as an interim measure until such time that immunoassays can be better harmonized through the use of an International Standard Serum.

## INTRODUCTION

Shigellosis is caused by the gram-negative bacteria of the genus *Shigella* comprising the following four species: *Shigella flexneri*, *Shigella sonnei, Shigella boydii,* and *Shigella dysenteriae. S. sonnei* and *S. flexneri are* responsible for the largest number of cases worldwide (1). The global disease burden is high, with an estimated 148,202 deaths due to shigellosis in 2019, more than half of which were in children under 5 years of age (2, 3). Disease management consists largely of supportive oral rehydration therapy, and antibiotic treatment is recommended when patients present with dysentery or have a laboratory-confirmed diagnosis of shigellosis, although this is complicated by the increasing emergence of antimicrobial-resistant *Shigella* isolates, including extensively drug-resistant isolates in some areas (4). As such, there is a strong public health need for effective *Shigella* vaccines, and although there is currently no licensed vaccine widely available, a number of candidate vaccine products are in development, some in the late-stage clinical trials (5–7).

Predicting the likely effect of vaccines in early phase development is challenging, and determining a true efficacy value requires the vaccine to be administered to a large population with a high disease burden and compared with a placebo. Measurement of vaccine efficacy is often unfeasible due to the lack of disease in target populations. Therefore, correlates of protection (CoPs) are used throughout pre-clinical and clinical development to predict the ability of the vaccine to generate an appropriate protective immune response. Ideally, a mechanistic correlate of protection (mCoP) directly measuring a functional response is employed (neutralization, opsonization, or bactericidal assays); however, such assays are often laborious and difficult to replicate between laboratories. The alternative is to employ a non-mechanistic correlate of protection (nCoP) that may act as a surrogate and is generally more feasible to measure than an mCoP. In some examples, measuring the amount of antigen-binding IgG may equate to a protective response (8), as IgG is commonly the initiating step of the active response, and for injected vaccines, it acts as an indicator of stimulation of the systemic adaptive immune response.

Serum IgG specific for *Shigella* lipopolysaccharide (LPS) has been proposed as a correlate of protection against shigellosis, the identification of which will facilitate vaccine development and licensure (9). Sero-epidemiological studies conducted in Israel revealed that pre-existent serum IgG antibodies to *S. sonnei* or *S. flexneri 2a* lipopolysaccharide were strongly associated with resistance against homologous *Shigella* spp. infection (9). These findings constituted key support to the glycoconjugate *Shigella* vaccine approach and the construction of the detoxified O-Specific Polysaccharide (O-SP)-Protein *Shigella* glycoconjugates by John Robbins and Rachel Schneerson at the US National Institutes of Health (10, 11). A recent study, which reanalyzed data from the two randomized controlled vaccine efficacy (VE) trials involving the detoxified *S. sonnei* O-SP covalently linked to the recombinant exoprotein A of *Pseudomonas aeruginosa* glycoconjugate (*S. sonnei*-rEPA), further highlighted the relevance of anti-LPS IgG as a correlate of protection. The study identified a protective threshold (expressed as an ELISA endpoint titer) of ≥1,600 anti-*S*. *sonnei* LPS IgG at day 17 post-vaccination, associated with a reduced risk of *S. sonnei* shigellosis and a predicted VE of 73.6% (95% CI: 65%−80%) (12). Moreover, the strong correlation between the vaccine-induced IgG anti-LPS titers and serum bactericidal activity (SBA) titers (r = 0.9; *P* < 0.01) indicated the functional capability of the IgG-anti-LPS ELISA-measured antibodies (12). The data from two different clinical trials were analyzed—one in young adults where the antibody measurements were expressed as endpoint titers (13), and one in children (14), where the antibody measurements were expressed in ELISA units (EU) relative to an internal reference serum. This difference highlights a wider challenge with identifying thresholds for correlates of protection, in that different laboratories use different assay protocols and may also report results for antibody measurements in different ways (e.g., endpoint titers vs. ELISA units), making comparison of data across clinical trials extremely difficult (9, 15). Therefore, there is a need to improve measurement standardization of anti-*Shigella* LPS antibody responses through the use of an International Standard Serum and more harmonized assay protocols. Several efforts are ongoing to achieve these goals to help accelerate the development and licensure of *Shigella* vaccines.

The GSK Vaccines Institutes for Global Health (GVGH) has developed a *S. sonnei* vaccine (1790GAHB) based on Generalized Modules for Membrane Antigens (GMMA) technology in which outer membrane vesicles released by bacteria are genetically modified to enhance blebbing (16). *Shigella* GMMA display LPS as the active moiety and contain lipid A genetically modified to reduce endotoxicity (17) while also inducing immune responses to outer membrane and periplasmic proteins (18). *S. sonnei* vaccine 1790GAHB (19) has been tested in five different clinical trials, which have shown the vaccine to be well tolerated and able to induce bactericidal anti-LPS IgG but failed to demonstrate clinical efficacy against shigellosis in a controlled human infection model (CHIM) in US adults (20, 21). However, by analysis of several immunological endpoints, it was possible to demonstrate that the presence of anti-*S*. *sonnei*-specific antibodies and their serum bactericidal activity correlate well with a reduced risk of shigellosis (22). Collectively, the results obtained with *Shigella sonnei* GMMA vaccine paved the way for the development of a next-generation 4-component vaccine consisting of GMMA derived from *S. sonnei* and *S. flexneri* 1b, 2a, and 3a (altSonflex1-2-3) (23). A Phase I/II observer-blind, randomized, controlled, multi-country study is currently ongoing (NCT05073003). Stage 1 of this trial, conducted in European adults, demonstrated the safety of the vaccine and the ability to induce functional serotype-specific immune responses (24).

To aid interpretation of the immunogenicity results obtained from this trial (and other trials where anti-*S*. *sonnei* LPS IgG is measured), a bridging study has been performed using a panel of human serum samples to identify the anti-*S*. *sonnei* LPS IgG level that corresponds to the threshold titer of 1,600 defined by Cohen *et al*. (12). This analysis has been performed using two ELISA methods used by other laboratories: the GVGH ELISA, which reports antibody response in EU/mL against an internal reference serum, and the Walter Reed Army Institute of Research (WRAIR) ELISA, which reports endpoint titers. Both ELISA methods will be used for the measurement of anti-*S*. *sonnei* LPS IgG responses in serum collected in ongoing clinical trials. The results of the study presented here will aid in immunobridging results from the Tel Aviv University (TAU) ELISA with the more recent clinical studies, allowing a more direct comparison of immunogenicity across vaccines and different clinical trials.

## MATERIALS AND METHODS

### Study samples

A panel of 32 human serum samples, covering a range of anti-*S*. *sonnei* LPS IgG levels, was provided by the Walter Reed Army Institute for Research (WRAIR) under a human use protocol (WRAIR 2415). The samples were obtained from a dose-finding challenge study in healthy adults, using *S. sonnei* strain 53G (19). To ensure that the study samples covered a wide range of anti-*S*. *sonnei* LPS IgG levels, the samples in the panel were selected based on results obtained in pre-study testing at WRAIR. Each laboratory was instructed to test all 32 samples using its internal ELISA protocol. For TAU and GVGH, three independent ELISA runs, on different days, were performed. Data from WRAIR are the endpoint titers from two ELISA runs. All samples were blinded by the sample coordinator (WRAIR) prior to shipment to each testing laboratory.

### ELISA antigen

In the WRAIR ELISA, LPS from *Shigella sonnei*, strain Moseley, was extracted using the Westphal procedure (25). The purified LPS product was lyophilized and stored at 22 ± 2°C in a desiccator. Prior to use in the ELISA, the *S. sonnei* LPS is suspended at 10 mg/mL in sterile water for injection (USP) and identity confirmed by western blot analysis using a *S. sonnei* LPS-specific monoclonal antibody (28.G8), a silver stained gel to confirm LPS banding pattern comparable with a reference standard, BCA to determine protein contamination (20 µg/mg of LPS), and SDS-PAGE with Coomassie staining (no protein bands identified after loading gel with 100 µg LPS).

The LPS coating antigen used by GVGH and TAU was extracted from outer membrane vesicles of *S. sonnei* 53G Δ*tolR virG::nadAB* strain (19), adapting the Westphal procedure (25). Purified LPS is stored at 4 ± 2°C and quantified by HPAEC-PAD analysis (26). Identity was confirmed by western blot using an *S. sonnei* LPS-specific monoclonal antibody (Inbios).

### ELISA methods

All three laboratories used an in-house ELISA protocol. Table 1 provides details of critical reagents used and the key features of each assay. The protocol used by TAU, with minor modifications, was used in all prior studies measuring the serum IgG antibody response to *Shigella* LPS following natural infection or vaccination with *Shigella* candidate vaccines. Control sera (pre- and post-vaccination or culture-proven infection) were used over the years to ensure consistency of results when the LPS or other reagent lots were modified.

**TABLE 1:** Critical reagents and key features of *S. sonnei* LPS-specific serum IgG ELISA methodologies used in the study^*a*^

Methodology/Reagent/Conditions	TAU	GVGH	WRAIR
ELISA plates	Flat bottom (Corning-Costar 3590)	Round bottom (Nunc Maxisorb)	Round bottom (Immulon 1b)
Antigen coating			
LPS conc.	0.5 µg/mL	0.5 µg/mL	10 µg/mL
Buffer	Carbonate buffer, pH 9.6	PBS	Carbonate buffer, pH 9.6
Time/Temp	1 h at 37°C	16 h at 2–8°C	16 h at 2–8°C
Blocking buffer	0.5% BSA and 0.5% Casein	5% fat-free milk in PBS	2% casein
Wash buffer	PBS with 0.05% Tween 20	PBS with 0.05% Tween 20	PBS with 0.05% Tween 20
Primary Ab: Time/Temp/Diluent	Overnight at room temperature in blocking buffer	2 h at 25 ± 2°C in blocking buffer	2 h at 22 ± 2°C in blocking buffer
Secondary Ab			
Vendor	AP conjugated anti-human IgG (KPL, 5220-0348)	AP conjugated anti-human IgG (Sigma, A3187)	Reserve AP labeled goat-anti-human IgG, gamma-chain specific conjugated secondary antibody (SeraCare, 5220-0348)
Dilution/Conc.	1:5,000	1:9,000 (corresponding to 0.3 µg/mL for the lot used)	1 µg/mL
Buffer	0.5% BSA and 0.5% Casein	PBS with 0.05% Tween 20 and 0.1% BSA	2% casein
Time/Temp	Overnight at room temperature	1 h at 25 ± 2°C	1 h at 22 ± 2°C
Substrate: Vendor/conc.	Para-nitrophenyl phosphate one component, ready-to-use substrate solution (SouthernBiotech, 0421-01L), 15 min at room temperature	1.0 mg/mL of para-nitrophenyl phosphate substrate in 0.2 M Tris buffer (Sigma, N2770), 1 h at 25 ± 2°C	1 mg/mL para-nitrophenyl phosphate (Sigma, P4744) in 10% diethanolamine buffer, 30 min at 22 ± 2°C
Data analysis/endpoint determination	The results were expressed in endpoint titers (last serum dilution yielding OD of 0.2 or higher)	EU/mL were determined as the average EU/mL of triplicate sera dilutions, selected from the dilution in which OD values fell within the limits of standard curve accuracy	The results were expressed in endpoint titers (last serum dilution yielding OD of 0.2 or higher)

^*a*^ Abbreviations used in table: AP (Alkaline Phosphatase); BSA (Bovine Serum Albumin); EU (Elisa Units); OD (Optical Density); PBS (Phosphate Buffered Saline).

### Reporting of data and statistical analysis

All raw data were returned to the MHRA study coordinator for independent analysis. For TAU and WRAIR assays, Geometric Mean (GM) endpoint titers, and for GVGH assays, mean ELISA Unit (EU)/mL estimates, taken across the independent ELISA runs, were used as final results for analysis. The results were log_10_-transformed for all calculations. Orthogonal regression (Deming with error variance ratio 1) was used to model a linear relationship between the results for each laboratory pair. Analysis was performed using Minitab software (version 18.1). To calculate 95% confidence intervals for predicted values, the R package “mcr” was used.

## RESULTS

The samples in the study panel covered a range of anti-*S*. *sonnei* LPS IgG levels and the estimates (geometric mean endpoint titers or mean EU/mL) for each of the serum samples assayed independently at the three laboratories using in-house protocols are shown in Table 2. In pre-study testing at WRAIR, eight of the samples returned results that were at or below the limit of quantification (LOQ). When tested again using the WRAIR ELISA, as part of this study, four of these samples were again identified to be at or below LOQ, and four returned low but detectable titers. Almost all (7/8) of these samples were below LOQ for the GVGH assay. In contrast, low but detectable titers were reported for all of these 8 samples using the TAU ELISA. None of the eight were included in the laboratory pair regression analysis, and they are not shown in Table 2. One sample (subject 167, day 7) returned a result below LOQ in the GVGH ELISA and low but detectable titers in the WRAIR and TAU ELISAs. This sample was included in the pairwise regression analysis and was assigned a value of half the LOQ as the GVGH result. This is marked with a single asterisk in Table 2. In addition, two samples were excluded as outliers from lab-pair regression analysis involving WRAIR because of large standardized residual values outside the range [−3,3] when initially fitting the regression models for WRAIR against data sets for both receiving labs (TAU and GVGH). These samples are marked with a double asterisk in Table 2.

**TABLE 2:** Summary of ELISA results from each of the three participating laboratories

Subject	Day	Expected antibody level based on pre-study testing at WRAIR	WRAIR titer	TAU titer	GVGH EU/mL
006	7	Low	800	400	212
020	7	Low	566	800	109
037	7	Low	800	1600	453
042	7	Low	800	635	197
046	7	Low	400	100	16
075	7	Low	800	504	137
099	7	Low	200	400	69
167	7	Low	141	200	6^*a*^
033	7	Medium	1,600	1,600	455
033	28	Medium	1,600	1,270	512
037	14	Medium	3,200	3,200	1,743
075	56	Medium	9,051	6,400	2,342
099	28	Medium	4,525	1,600	482
107	7	Medium	4,525	4,032	995
135	28	Medium	4,525	3,200	1,162
137	7	Medium	6,400	3,200	890
045	28	High	36,204	12,800	5,107
045	56	High	36,204	8,063	2,603
060	28	High	36,204	10,159	4,227
075	28	High	18,102	12,800	4,143
107	56	High	12,800	12,800	3,281
137	14	High	51,200	12,800	5,177
165	28	High	36,204^*b*^	1,270	188
165	56	High	51,200^*b*^	800	122

^*a*^ Sample was assigned half the LOQ for regression analysis.

^*b*^ Samples with large standardized residual values outside [−3,3] range when initially fitting the regression models for the coordinator lab (WRAIR) against data sets for both receiving labs (TAU and GVGH) were excluded from further analysis involving the WRAIR assay.

A comparison of assay precision based on data obtained in this study is given in Table 3, showing the percentages of cases where titers from independent assay runs did not differ, where they differed by 2-fold or less, or where they differed by 4-fold or less. Taking a definition of precision as the percentage of cases not differing by more than 2-fold, as this dilution spacing was used for all assays, the WRAIR, TAU, and GVGH assays gave precision values of 96%, 100%, and 100%, respectively.

**TABLE 3:** Assay precision data for ELISAs performed by WRAIR, TAU, and GVGH

Difference in repeat endpoint titers or EU	WRAIR	TAU	GVGH
None (equal)	50.0%	80.6%	N/A^*a*^
≤2-fold	95.8%	100.0%	100.0%
≤4-fold	100.0%	100.0%	100.0%

^*a*^ N/A, not applicable.

**Fig 1:**
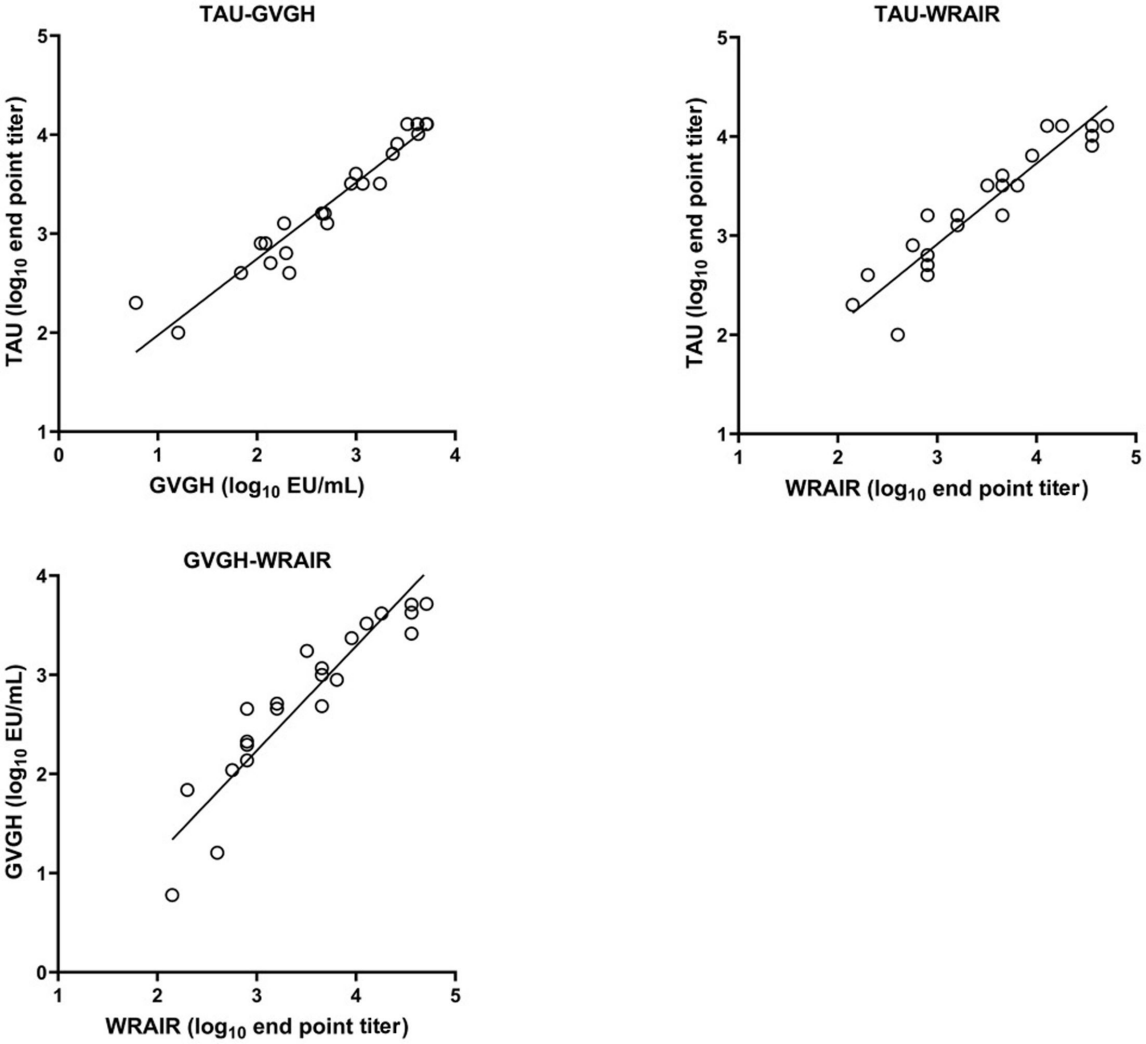
Comparison of results from WRAIR, TAU, and GVGH for each laboratory pair. Only data used for regression analysis are plotted.

A comparison of results for each laboratory pair is shown in Fig. 1. Observed correlations between laboratory pairs are summarized in Table 4. The highest degree of correlation was observed between TAU and GVGH with Pearson r = 0.962 (*P* < 0.001), whereas correlations for both TAU and GVGH with WRAIR gave Pearson r = 0.935 and 0.927, respectively (*P* < 0.001).

**TABLE 4:** Correlation between laboratory pairs

Laboratories	Pearson r	Spearman ρ	n
TAU – GVGH	0.962	0.954	24
TAU – WRAIR	0.935	0.944	22
GVGH – WRAIR	0.927	0.968	22

**Fig 2:**
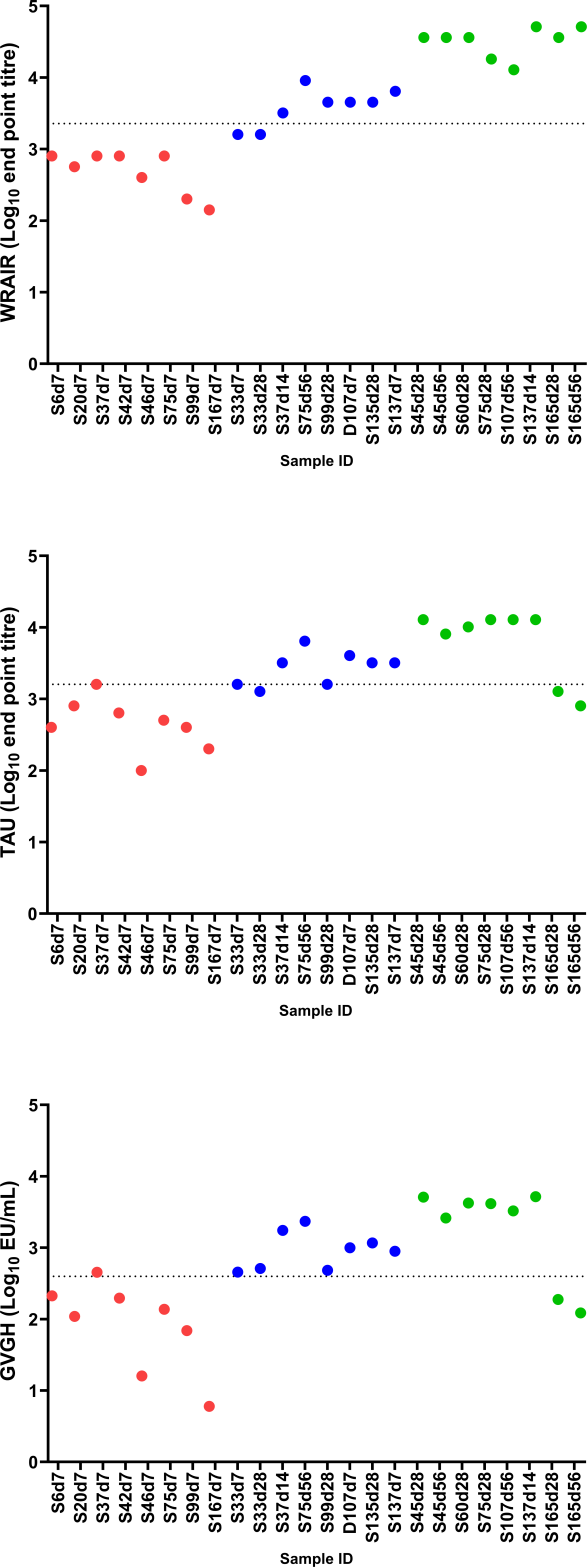
Responses for the individual serum samples for each laboratory ordered as listed in Table 2. Color coding indicates samples that were expected to have low (red), medium (blue), or high (green) anti-*S*. *sonnei* LPS IgG levels. Dotted lines indicate the titer of 1600 (for the TAU data set) and its corresponding value in the other two data sets based on the regression analysis.

Using the fitted equation from the regression analysis to convert a TAU titer of 1,600 (i.e., 3.204 log_10_ titer) to GVGH EU/mL gives an estimate of 396 EU/mL (i.e., 2.597 log_10_ EU/mL). A 95% confidence interval for this estimate is calculated to be 315–497 EU/mL. For the WRAIR assay, a TAU titer of 1,600 corresponds to an endpoint titer of 2,276 with a 95% confidence interval of 1,662–3,118. The fitted equations for each laboratory pair are shown in Table 5 along with the result that corresponds to a TAU titer of 1,600. Fig. 2 shows the reported results for each serum sample in the low, medium, and high categories, ordered as listed in Table 2, and also indicates the TAU titer of 1,600 (for the TAU data set) and its corresponding value in the other two data sets based on the regression analysis shown in Table 5.

**TABLE 5:** Fitted equation from regression analysis and predicted ELISA titers that correspond to a TAU titer of 1,600

Laboratory pair	Fitted equation^*a*^	Result corresponding to TAU titer of 1600^*b*^
TAU and GVGH	GVGH = −1.545 + 1.293 TAU	396 (315–497) EU/mL
TAU and WRAIR	WRAIR = −0.573 + 1.227 TAU	2276 (1662–3118) Endpoint titer
GVGH and WRAIR	WRAIR = 0.869 + 0.952 GVGH	N/A^*c*^

^*a*^ Equation uses log_10_-transformed titers.

^*b*^ Predicted titer and 95% confidence limits calculated from fitted regression model.

^*c*^ N/A, not applicable.

## DISCUSSION

The availability of International Standards for antibody assays (established by the World Health Organization) provide the basis for standardization of these assays, such that antibody responses can be reported in a common unit (the International Unit) (27). This facilitates the comparison of data across laboratories and between different trials. However, international standards for antibody assays are not yet available for many vaccines that are currently in clinical development. As a result, the measurement of antibody responses in clinical samples from a number of vaccine trials cannot be easily compared–since laboratories will employ different local standards, often reporting their results in different units entirely (e.g., endpoint titers vs. ELISA Units/mL).

The ability to reliably compare data across different laboratories and clinical trials conducted at different times is even more important when the responses being measured have been identified as a CoP (or putative CoP). The complexity of the human immune response is a challenge for the identification of a CoP, and although it is accepted that there is no single immune factor that determines protection against an infectious disease, the identification of major responses that are easily measurable has enormous value in vaccinology (28). A harmonized measurement system, with results that are traceable to a primary standard (such as a WHO International Standard), will facilitate identification of a threshold level for an immune correlate that is indicative of protection, as in the case for diphtheria, tetanus, hepatitis A, hepatitis B, measles, pneumococcal, and meningococcal vaccines (29).

Ideally, an mCoP directly measures a functional immunological parameter responsible for protection (e.g., neutralization, opsonization, or bactericidal assays); however, such assays are less amenable to high-throughput analysis for large numbers of clinical samples, and they are technically demanding (requiring containment facilities in some cases). The alternative is to measure the amount of binding antibody (typically IgG) as an nCoP that equates to a protective response, as IgG is commonly the initiating step of the active immune response and, for injected vaccines, an indicator of stimulation of the adaptive immune response. In the case of protection against shigellosis, a correlation between anti-LPS IgG levels and protection against disease has been demonstrated both in vaccinated subjects and in subjects previously infected with *Shigellae* (9, 12). Although work is ongoing to understand the mechanisms involved in preventing or clearing *Shigella* infections, levels of anti-LPS serum IgG provide a strong predictor of protection and are easily measurable in high-throughput immunoassays.

There are numerous vaccines under development targeting shigellosis; however, with disease attributable across four different *Shigella* species encompassing more than 50 O-antigen serotypes, development of a broadly protective vaccine has proved challenging (5). The vaccines developed to date can be categorized as whole cell (killed and attenuated) vaccines and subunit vaccines. Balancing immunogenicity and reactogenicity coupled to inducing a broadly cross-protective immune response has hindered the development of whole-cell vaccines (5), and currently, the subunit vaccines are the most advanced in clinical evaluation. Glycoconjugate vaccines based on the O-antigen portion of the LPS from *Shigella* reduce the risk of reactogenicity and can be formulated with multi-valency to offer broader protection to key disease-causing strains (30). An alternative sub-unit vaccine is the GMMA outer membrane vesicle platform used to produce a four-valent vaccine encompassing outer membrane vesicles from genetically modified *Shigella* strains. These bacteria have been engineered to reduce reactogenicity of the LPS and increase the vesiculating phenotype of the parental bacterial strain while the structure of the O-antigen is maintained (20, 23).

*Shigella* vaccine development is a good example of a field where an International Standard Serum for antibody assays is not yet available, and therefore, the ability to compare data from different laboratories and different clinical trials is more difficult. Data obtained from *S. sonnei* vaccine efficacy studies have identified the protective IgG threshold value as being an end-point titer of ≥1,600, or 6.6 ELISA units (EU) in adults, predicting 73.6% VE, and an end-point titer of ≥1072 or 4.5 EU in children aged 3−4, predicting 63% VE (12–14). The EU was defined from a standard reference serum prepared at the U.S. National Institutes of Health from convalescent sera of *S. sonnei* shigellosis patients and assigned an arbitrary unit of 100 EU (12). The serum utilized is no longer available, and thus, it is difficult to compare the readout of recent trial serum to that of the earlier studies. Recently, there has been a rejuvenated interest in the development of *Shigella* vaccines, with several candidates in development and entering clinical trials. An understanding of the performance of the novel vaccines compared with those from the 1990s is important to provide a benchmark for the developers to ensure the new vaccines are non-inferior to previous formulations.

GVGH has tested the *S. sonnei* GMMA-based vaccine in a series of clinical trials (20). The group developed an internal standard serum, and an arbitrary value of 1 EU/mL was assigned from the reciprocal of the dilution giving a reading of OD 1 in the GVGH ELISA. Of note is that the EU assigned in Israel in the 1990s and the EU assigned by GVGH in the 2020s are unrelated and cannot be directly compared. In the bridging study presented here, the 1,600 protective endpoint titer of the TAU study corresponds to a GVGH value of 396 EU/mL (95% CI: 315–497), and an endpoint titer of 2276 (95% CI: 1,662–3,118) in the WRAIR ELISA as the protective values. Sourcing large numbers of human serum samples with a wide range of anti-*Shigella* antibody titers, and with sufficient volume for a multi-laboratory study, is challenging, and the data presented here provide valuable information on how different immunoassays compare. The panel of samples used in this bridging study is relatively small, and the authors acknowledge that using a larger serum panel that is representative of the full range of antibody titers would improve the robustness of any conclusions regarding lab-to-lab comparisons and including a greater number of samples around the TAU endpoint titer of 1,600 would allow for the calculation of corresponding cutoff values with higher precision. Further studies, involving serum from trials conducted in different settings and in different age groups, will also enable more robust conclusions regarding bridging of threshold values between different ELISA methods.

Taking a definition of precision as the percentage of replicate titers not differing by more than 2-fold as done elsewhere (31), the WRAIR, TAU, and GVGH assays gave precision values of 96%, 100%, and 100%, respectively, based on the results obtained for this panel of serum samples. Given the assay precision, it is consistent with assay expectations that some samples with low antibody levels may give measurable titers in one assay and be below the limit of quantitation when tested again in the same assay.

Overall, the results for each laboratory pair show excellent correlation. The results returned for two samples (subject 165, day 28 and day 56) were not consistent in terms of the profile for how results in the GVGH and TAU ELISAs compared with the WRAIR ELISA, as illustrated by the large standardized residual values for these samples. Within each laboratory, 100% precision was observed for these two samples across the three (GVGH and TAU) or two (WRAIR) assay runs. An excellent correlation between laboratory pairs was seen across the rest of the study panel, including samples with high titers. The striking discrepancy observed for these two samples between the WRAIR ELISA and the other two laboratories cannot be explained and was considered to be outliers for the WRAIR data set and therefore excluded from the regression analysis involving that data set.

There are three ambiguous data points with respect to concluding whether that sample is below or above the assay threshold based on the conversion according to results in this study: subject 33 day 28, which is below the protective threshold in the TAU ELISA and WRAIR ELISAs but above the threshold in the GVGH ELISA; subject 33 day 7 and subject 37 day 7, which are below the protective threshold in the WRAIR ELISA but at or above the threshold in the GVGH and TAU ELISAs. These three samples are at or close to the protective threshold of 1,600 in the TAU ELISA, and, with the precision of the assay considered, some discrepancies in conclusion regarding protection status for a given sample would be expected between the laboratories. Indeed, the nature of these assays and their achievable precision would not allow the definition of a single cutoff value that leads to the same outcomes (i.e., above or below the cutoff) in all assays for samples with titers close to the defined cutoff value. The definition and use of any equivalent cutoff value must be carefully considered, taking into account the assay precision and the potential need for equivocal ranges around such values.

### Conclusion

The use of appropriately standardized immunoassays with results reported that are traceable to a primary standard offers the best platform for comparing data and making evidence-based decisions (such as a threshold value for an identified CoP). In fields that have not yet reached this level of standardization maturity, comparability studies such as the one presented here offer an alternative solution. Our bridging study has demonstrated excellent correlation of the ELISA data between the laboratories, with the majority of samples assigned to the same category of protective antibody concentration or non-protective antibody concentration. The analysis presented here provides a way to meaningfully compare results obtained with clinical samples across the three ELISAs used by the three participating laboratories, using the fitted equation from the regression analysis to better understand clinical data generated by these laboratories in vaccine or seroepidemiology studies. Methodological differences in the ELISAs performed by the three labs were not accounted for during data analysis, since our intention was to highlight how difficult it is to compare the outputs from labs performing assays that differ in methodology and readout. Given the variety of ELISA methods utilized in the Shigella vaccine development field, due in part to a previous lack of global harmonization, it was important to evaluate how an endpoint titer determined in the ELISA used by TAU related to titers achieved using other methodologies, in the context of several ongoing or recently completed age-descending Shigella vaccine clinical studies conducted in target populations, to better facilitate result comparisons across these studies. Several of these clinical studies (NCT04056117, NCT04602975, NCT06663436, and NCT05073033) utilized either the WRAIR or GVGH ELISA methodologies to determine serum IgG responses directed to Shigella LPS, and therefore, the ability to immunobridge to endpoint titers determined in the TAU ELISA is a critical decision component for future development activities. Moving forward, the MHRA is developing a candidate WHO International Standard Serum for use in *Shigella* antibody assays in a project funded by the Gates Foundation. Once available, the International Standard Serum will facilitate wider harmonization of *Shigella* immunoassays and support the assessment of the next generation of *Shigella* vaccines through the developmental and regulatory pipeline to clinical use in the populations currently affected by shigellosis (15, 32).
